# Supplementary material for: Building an annotated corpus for automatic metadata extraction from multilingual journal article references
Source: PLoS One. 2023 Jan 20;18(1):e0280637. doi: 10.1371/journal.pone.0280637 (PMC9858828; doi:10.1371/journal.pone.0280637)
Supplement: Attachment — Submitted filename: Response to Reviewers.doc [file pone.0280637.s002.doc]

**Original Manuscript ID:** PONE-D-22-06759

**Original Article Title: “**Building an annotated corpus for automatic metadata extraction from multilingual journal article references”

**To:** PLOS ONE Editor

**Re:** Response to reviewers

Dear Editor,

Thank you for allowing a resubmission of our manuscript titled **“**Building an annotated corpus for automatic metadata extraction from multilingual journal article references.” We appreciate the time and effort dedicated by you and the reviewers in providing your valuable feedback on the manuscript. We have incorporated changes to reflect the suggestions provided by the reviewers. We hereby submit (a) our point-by-point response to the comments (below) (response to the editor and reviewers), (b) an updated manuscript with red highlighting indicating the changes, (c) a clean, updated manuscript without highlights (PDF main document), (d) image files for newly created figures (Fig8 and Fig9), and (e) a supporting excel file (S1 File).

Best regards,

Hyesoo Kong

**Journal Requirements (response to the editor):**

**When submitting your revision, we need you to address these additional requirements.**

**#1.** Please ensure that your manuscript meets PLOS ONE's style requirements, including those for file naming. The PLOS ONE style templates can be found at

https://journals.plos.org/plosone/s/file?id=wjVg/PLOSOne_formatting_sample_main_body.pdf and

https://journals.plos.org/plosone/s/file?id=ba62/PLOSOne_formatting_sample_title_authors_affiliations.pdf

***Author response:*** *Thank you. We have tried to adhere to this format in our revised paper. We will be glad to make further changes if we missed any formatting instructions.*

**#2.** Thank you for stating in your Funding Statement:

“This research was supported by the Korea Institute of Science and Technology Information (KISTI) of the Ministry of Science and ICT, South Korea (MSIT) (No. K-22-L01-C01-S01: Construction on Intelligent SciTech Information Curation). The funders had no role in study design, data collection and analysis, decision to publish, or preparation of the manuscript.”

Please provide an amended statement that declares *all* the funding or sources of support (whether external or internal to your organization) received during this study, as detailed online in our guide for authors at http://journals.plos.org/plosone/s/submit-now. Please also include the statement “There was no additional external funding received for this study.” in your updated Funding Statement.

Please include your amended Funding Statement within your cover letter. We will change the online submission form on your behalf.

***Author response:*** *We included *all* the funding or sources of support. This will be move to the cover letter as mentioned by the academic editor.* *We have now amended the funding information as follows. The amended statement reads* ***“This research was supported by the Korea Institute of Science and Technology Information (KISTI) of the Ministry of Science and ICT, South Korea (MSIT) (No. K-22-L01-C01-S01: Construction on Intelligent SciTech Information Curation). The funders had no role in study design, data collection and analysis, decision to publish, or preparation of the manuscript. There was no additional external funding received for this study.”***

**Response to the reviewers:**

**Comments from Reviewer #1**

**Reviewer#1, comment #1:** Is locating the references strings in journal articles a problem? If so, is your data helpful in solving it?

***Author response:*** *Thank you for the comment. We have collected, stored and managed all journal articles in PDF format. Therefore, it is necessary to find and extract the list of references included in the PDF before the metadata extraction process starts. We have been collecting journal articles from over 500 domestic journals annually. Because each journal has a different design of the paper and reference numbering style, it is difficult to automatically locate and extract the list of references in journal articles. Therefore, we have manually extracted them thus far. The corpus mentioned in this paper is built to extract metadata automatically under the assumption that a single reference is given. Recently, we started a study aimed extracting the bibliography list from pdf papers automatically. In more detail, we plan to construct an image-based corpus for detecting the locations of references and for separating into individual references. Using this corpus, we will train the object detection models such as YOLO. For extracting reference texts from the detected objects, we will use the OCR technologies. In the near future, we plan to fully automate the bibliographic metadata extraction process.*

**Reviewer#1, comment #2:** You state that previous methods of parsing reference strings do not perform sufficiently well to be used without human intervention. Does your BERT model produce results good enough to be used without a human examining results? Are you using it in your curation process for your KISTI database?

***Author response:*** *Thank you for the pertinent comment. The journal articles collected by KISTI include many non-English references and English. For these non-English references, existing methods did not correctly extract their metadata. On the other hand, as mentioned in the paper, the metadata extracted by BERT-REF1 were almost identical to the strings of answer metadata verified by humans. In addition, after we conducted this research, we continuously accumulated the results of both the manual extraction of metadata and the automatic extraction using BERT-REF1 for several months. In conclusion, it was found that BERT-REF1 produced sufficiently good results to be used without human intervention. Currently, the metadata for given references are automatically extracted without any human intervention and accumulated in the KISTI DB. Therefore, BERT-REF1 is now applied to fully automate the metadata extraction of references during the curation process for the KISTI Database.*

**Reviewer#1, comment #3:** p.12, line 183 “each group delicately examined” would be better “each group carefully examined.”

***Author response:*** *Thank you for the comment. We have corrected the term accordingly.*

***Author action:*** *We changed the term “delicately” to “carefully.”*

***(line 182, page 6)*** *They were then paired, and two annotators in each group carefully examined the same pre-labeled references.*

**Comments from Reviewer #2**

**Reviewer#2, comment #1:** All assessment scenarios have better results (Tab 10, 11, and 12). In this sense, doubts about the work results can be pointed out. One suggestion is to propose an extra analysis to validate the results through textual data visualization techniques. For example, agglomerative clustering methods can be proposed to visualize the results.

***Author response:*** *Thank you for the suggestion. Tables 10, 11, and 12 show the accuracies calculated by the annotators manually comparing the answer metadata with the metadata predicted by GROBID and BERT-REF1. Therefore, questions concerning the the results can arise because they result from manual evaluation by annotators. Therefore, we attempted to perform an additional analysis to validate the results through textual data visualization techniques such as agglomerative clustering. However, we found it challenging to visualize the validation results using clustering methods because our experiment, as shown in Tables 10, 11, and 12, simply aimed to verify equality between the answer metadata and the predicted metadata by each model. Therefore, we performed another experiment that automatically compares the string similarities between the answer metadata and the predicted metadata by each model.*

*In the experiment, we automatically calculated the string similarities between the answer metadata and the metadata predicted by BERT-REF1 and GROBID using the Levenshtein distance. The answer metadata for the 12,887 references mentioned in the paper can be considered accurate and reliable since the KISTI database has been regularly evaluated by the Korea Data Agency, a government-affiliated organization, to check the DB quality, and the administrator of this research additionally inspected them. Thus, it can be considered that the higher the similarity with the answer metadata is, the better the model performance. Figure 8 shows the similarities according to the metadata field types. Similar to the manual evaluation results shown in Tables 10, 11, and 12, BERT-REF1 slightly outperformed the similarity performance of GROBID for the English references. However, for non-English references, BERT-REF1 showed significantly better performance than GROBID. In particular, it was found that the similarity performance between the two models for author names, titles, and journal names in non-English references was significantly different. We also visualized the similarity difference between BERT-REF1 and GROBID for the above three metadata field types in 3,348 non-English references, as shown in Figure 9. In Figure 9, the red similarity points of metadata predicted by BERT-REF1 were distributed closer to 1.0 than GROBID. This is because GROBID could not extract accurate metadata from non-English references. Based on this experiment, we concluded that BERT-REF1 outperformed GROBID not only in English references but also in non-English references. We also added Excel sheets containing the answer metadata and the metadata predicted by BERT-REF1 and GROBID for all 12,887 references as supplementary information.*

***Author action:*** *We added the following paragraphs to the method and result section.*

***(line 354, page 10)*** *“We periodically analyzed the patterns of data errors in the KISTI database in terms of several assessment areas, including column consistency and uniqueness according to the database quality certification-value(DQC-V) of the Korea Data Agency, a government-affiliated organization aimed at building the circulation of a data ecosystem. In the analysis, the platinum class certification, which is granted only when the accuracy of data values in the database is 99.97% or higher, was obtained. Despite the analysis, some metadata field values associated with the 12,887 references could be incorrectly entered. Thus, the administrator manually corrected such incorrect values by scanning each individual value. After completing all these steps, we obtained completely reliable data.”*

***(line 381, page 11)*** *"Unlike the aforedescribed manual verification method, we automatically calculated the string similarity between the answer metadata corresponding to the new data and the metadata predicted by the BERT-based model and GROBID by using the Levenshtein distance, which is a performance metric to determine the similarity between two strings– the source string (answer metadata) and the target string (predicted metadata by each model). Because the answer metadata were precisely verified by professional curators as aforementioned, the higher the similarity with the answer metadata, the better would be the model performance. Therefore, we analyzed the performance difference between the two models in predicting accurate metadata on the basis of this metric. The experimental results are detailed in the next section.”*

***(line 556, page 18)*** *“Lastly, we also calculated the string similarity between the answer metadata and the metadata predicted by BERT-REF1 and GROBID by using the Levenshtein distance. Fig 8 depicts the similarities according to the metadata field types. Notably, the closer the similarity is to 1, the higher would be the string similarity with answer metadata. In Fig 8, the first graph represents the results for all 12,887 references, and the others illustrate the results for 9,539 English references and 3,348 non-English references. Similar to the manual verification results shown in Table 11, BERT-REF1 slightly outperformed the similarity performance of GROBID for the English references. However, for non-English references, BERT-REF1 showed significantly better performance than GROBID. In particular, it was found that the similarity performances of the two models pertaining to author names, titles, and journal names were significantly different. For example, in BERT-REF1, the similarity of author name, title, and journal name was higher than GROBID by 0.7539, 0.8776, and 0.8477, respectively. Excel sheets containing the answer metadata and the metadata predicted by BERT-REF1 and GROBID for all 12,887 references are provided as supplementary information in S1 File.”*

*“Additionally, we visualized the similarity difference between BERT-REF1 and GROBID for the aforementioned three metadata field types in 3,348 non-English references as shown in Fig 9. In Fig 9, the red circles indicate the similarity values for the non-English references for BERT-REF1, and the black circles represent the corresponding similarity values for GROBID. As a result, for the three metadata field types, most of the red circles, representing the similarity of metadata predicted by BERT-REF1, were distributed close to 1.0. However, the black circles, indicating the similarity of metadata predicted by GROBID, were more likely to be distributed close to 0.0. This implies that GROBID could not precisely extract the metadata from given non-English references. The experimental results lead to the conclusion that BERT-REF1 outperforms GROBID in extracting metadata from references.”*

***(Figure 8, page 18)*** *Similarity performance of GROBID and BERT-REF1 for metadata field types based on the Levenshtein distance metric. The first graph shows the results for all 12,887 references; the rest represent the results for 9,539 English references and 3,348 non-English references..*

***(Figure 9, page 19)*** *Visualization of the similarity values and differences between BERT-REF1 and GROBID for the three metadata field types in 3,348 non-English references. The X-axis represents each non-English reference. The red circles indicate the similarity values for non-English references for BERT-REF1, and the black circles represent the corresponding similarity values for GROBID.*

***(S1 File, page 19)*** *The answer metadata and the metadata predicted by BERT-REF1 and GROBID for all 12,887 references to measure the similarity performances of the two models based on the Levenshtein distance.*
