# Supplementary material for: Building an annotated corpus for automatic metadata extraction from multilingual journal article references
Source: PLoS One. 2023 Jan 20;18(1):e0280637. doi: 10.1371/journal.pone.0280637 (PMC9858828; doi:10.1371/journal.pone.0280637)
Supplement: Attachment — Submitted filename: Response to Reviewers.doc [file pone.0280637.s003.doc]

Dear Dr Sanaa Kaddoura

Thank you for giving us the opportunity to submit a revised draft of my manuscript titled “Building an annotated corpus for automatic metadata extraction from multilingual journal article references” (manuscript ID: PONE-D-22-06759R1**)** to PLOS ONE. We appreciate the time and effort that you and the reviewers have dedicated to providing your valuable feedback on our manuscript. We have incorporated changes to reflect the suggestions provided by the reviewers and highlighted the changes within the manuscript. Lastly, we reviewed our reference list to ensure that it is complete and correct.

We hereby submit our point-by-point response to the comments (below) (response to the editor and reviewers). We look forward to hearing from you in regarding our submission and to respond to any further questions and comments you may have.

Sincerely,

Hyesoo Kong

Korea Institute of Science and Technology Information

Yuseong-gu, Daejeon-Si

Republic of Korea

[+82-10-6306-8303]

[+82-042-869-1669]

[hyesoo@kisti.re.kr](mailto:hyesoo@kisti.re.kr)

**[Comments from Editor]**

Please review your reference list to ensure that it is complete and correct. If you have cited papers that have been retracted, please include the rationale for doing so in the manuscript text, or remove these references and replace them with relevant current references. Any changes to the reference list should be mentioned in the rebuttal letter that accompanies your revised manuscript. If you need to cite a retracted article, indicate the article’s retracted status in the References list and also include a citation and full reference for the retraction notice.

***Author response:*** *In response to the above comments, we reviewed our reference list to ensure that it is complete and correct.*

***Author action:*** *We updated some of the references as follows:*

***(reference #3, page 19)*** *Pranckut˙e, R Web of Science (WoS) and Scopus: The titans of bibliographic information in today’s academic world. Publications. 2021; 9(1):12.* [*https://doi.org/10.3390/publications9010012*](https://doi.org/10.3390/publications9010012)

***(reference #5, page 19)*** *Khabsa M, Giles CL. The Number of Scholarly Documents on the Public Web. PLoS ONE. 2014; 9(5):e93949.* [*https://doi.org/10.1371/journal.pone.0093949*](https://doi.org/10.1371/journal.pone.0093949)

***(reference #18, page 20)*** *Sennrich R, Haddow B, Birch A. Neural machine translation of rare words with subword units. arXiv:1508.07909 [Preprint] 2016. Available from: https://arxiv.org/abs/1508.07909*

**[Comments from Reviewer 1]**

(No Response)

***Author response:*** *The reviewer 1 did not give any comment.*

**[Comments from Reviewer 3]**

The author (s) improved the paper based on the received comments. the paper can be published after improving language.

***Author response:*** *In response to the above comments, all spelling and grammatical errors pointed out by the reviewers have been corrected. We highlighted all the changes within the revised manuscript.*
